# Lessons from studies with murine cytomegalovirus that could lead to a safe live attenuated vaccine for human cytomegalovirus

**DOI:** 10.1099/acmi.0.000147

**Published:** 2020-06-22

**Authors:** Clive Sweet

**Affiliations:** ^1^​ School of Biosciences, University of Birmingham, Birmingham, UK

**Keywords:** codon preference, cytomegalovirus, mixed mutants, vaccine

## Abstract

Studies with a murine cytomegalovirus mutant *tsm5* suggested two possible approaches to producing a live attenuated human cytomegalovirus vaccine. One approach would be to use a combination of five to six mutants where an attenuating mutation in the gene of one mutant is compensated by the wild-type version in a second mutant, which in turn has a mutation in a different gene compensated by the wild-type version in a third mutant, etc. Important genes in this approach could include those involved in DNA replication. The importance of the carboxy terminase of the primase gene (M70/UL70) for its function suggested a second approach where some of the natural codons in this region could be substituted with synonymous non-preferred (minor) codons that would reduce the replication fitness of the mutant.

## Introduction

While human cytomegalovirus (HCMV), a member of the family β-herpesvirus, generally produces subclinical and persistent infection in healthy individuals, reactivation of latent infection can lead to severe disease in immunocompromised patients. Such infections may lead to multi-organ dysfunction and symptoms of hepatitis, enteritis, pneumonia and cerebrospinal infection [1].

In 20-year-old adult populations, about 60 % show seroconversion, but this increases with age. Horizontal transmission usually results from exposure to infected body fluids that mainly occurs during sexual activity or following contact with infected young children, but it may occur via blood transfusion or a transplanted organ. Vertical transmission occurs transplacentally or at the time of birth.

Three groups of immunocompromised hosts are susceptible to severe HCMV disease: (a) the immunologically immature foetus, (b) the transplant recipient as a result of anti-rejection treatment, and (c) AIDS patients with loss of adaptive immune responses. In addition to a vaccine being required for such compromised patients, a childhood HCMV vaccine could reduce transmission to both seronegative and seropositive mothers, while vaccination of adolescent and adult women could prevent transplacental transmission [2]. Thus, a vaccine to reduce the incidence and severity of infection and disease is a public-health priority.

### Current approaches to the development of a vaccine

Currently, despite being targeted as a high priority for vaccine development [3], there is no vaccine licenced for use against HCMV, but a number of experimental vaccines are being examined in animal models and in clinical trials [3–6]. It is anticipated that both humoral and cellular immunity needs to be stimulated to prevent acquisition of infection and/or for therapeutic improvement of disease. Various proteins are being investigated as vaccine candidates, including viral glycoproteins gB, gH, gN, gM, gO and gL, and unique long (UL) 128–131 as targets for eliciting neutralizing antibodies, and pp65 and immediate-early 1 (IE1) for T cells, either alone or in combination. These are being delivered as DNA vaccines, co-expressed in a viral vector or as mRNA molecules.

Generally, live attenuated vaccines are more successful for protection against viruses, as they induce both humoral and cellular immunity. Researchers have attempted to develop a live attenuated HCMV vaccine without success. Both the laboratory adapted AD619 and Towne strains stimulated both humoral and cellular responses, but failed to provide protection in transplant recipients or seronegative mothers. A notable recent success for a herpes virus is the live attenuated varicella-zoster virus (VZV) vaccine, which has a vaccine efficacy of 70–96 % in preventing chickenpox and is now recommended for prevention of shingles in the elderly [7, 8]. This vaccine virus has been passaged through human embryonic lung fibroblasts, guinea pig embryonic cells and two human diploid cell lines (WI-38 and MRC-5). It is a mixture of genotypically distinct VZV strains with at least 137 single nucleotide polymorphisms (SNPs). Six of these SNPs are ‘fixed’ and probably important for attenuation [9].

### Studies with murine cytomegalovirus (MCMV) mutant *tsm5*

MCMV is generally regarded as a good model for studying virus pathogenicity and is widely used for experimental vaccine development [5, 10]. Furthermore, MCMV genome organization and replication has many similarities with HCMV, the majority of their ORFs are collinear and 78 genes of the latter have significant amino acid identity with genes of HCMV; these ORFs are designated with an 'M', whilst genes unique to MCMV are designated ‘m’ [1]. Various vaccine strategies have been examined in this model, including immunization with recombinant plasmids expressing MCMV immunogens, peptide-based vaccines, live attenuated vaccines deleted of immune evasion genes and vectored vaccines expressing glycoproteins.

At Birmingham University, a panel of temperature-sensitive (*ts*) mutants was created by chemical mutagenesis of the K181 Birmingham variant of MCMV using *N*-methyl-*N*-nitro-nitrosoguanidine [11]. The *ts* mutants were selected for a restricted growth phenotype at the non-permissive temperature of replication of 39 °C and above. One of the mutants, *tsm5*, proved extremely interesting and was studied further. It was considerably restricted in its ability to form plaques (80- to 200-fold) and replicate (800- to 1200-fold) at 39 °C. Furthermore, attempts to produce mouse passaged virus were unsuccessful, indicating that *tsm5* was also attenuated *in vivo* [11–13].

Subsequent characterization demonstrated that *tsm5* did not produce detectable infectious virus in tissues of BALB/c mice at any time up to 60 days post-infection (p.i.). In contrast, *tsm5* DNA was detected in almost all tissues at all times up to 21 days p.i. and in salivary glands at 60 days p.i. Expression of immediate-early (IE1), early (E1) and late (gB) phase genes was detected in all tissues, except kidney, at some time up to 21 days p.i., but not at 60 days p.i. [14]. Following immunosuppression with cortisone acetate and anti-mouse lymphocyte serum, infectious virus was still not detectable, although *tsm5* DNA was detected and all three transcripts were produced in most tissues [13, 14]. Furthermore, immunization of BALB/c mice with as little as 40 plaque-forming units (p.f.u.) of *tsm5* protected mice against a sublethal dose of virulent salivary gland grown K181 Birmingham virus and no virus could be isolated from any tissue at 3–42 days after challenge. *tsm5* induced high-titre neutralizing antibody and a CD8^+^ cytotoxic T lymphocyte response. In severely compromised immunodeficient (SCID) mice, no infectious virus was detected at 14 days p.i., but by day 28 p.i. virus titres were from 10^2.5^ p.f.u. ml^−1^ in liver to 10^7.3^ p.f.u. ml^−1^ in salivary glands. *tsm5* virus recovered from tissues of SCID mice retained the *ts* phenotype [15]. These results suggested that low level virus replication proceeds in immunocompetent mice, but is limited by the host response.

NimbleGen Comparative Genome Sequencing (CGS) identified 10 synonymous and 15 non-synonymous SNPs in *tsm5*, and 14 of the latter were confirmed by conventional sequencing: m20 (2 SNPs), m21, M25, M34, M36, M47, M53, M70, M98, m132.1, m139, m141, m143 [16]. Two further SNPs (M27, M56) not identified by CGS were identified by sequencing, which also revealed that *tsm5* was a mixture of mutants in that at least six loci (M27, M36, M53, M56, M70, M98) were polymorphic, although generally the mutant nucleotide was dominant.

*tsm5* produced few capsids at 40 °C and these lacked DNA, probably because DNA synthesis was significantly reduced, although the DNA was processed similarly to that of the wild-type (wt) virus. Examination of genes involved in DNA packaging and cleavage (M52, M56, M98) and DNA replication (M70) revealed a mutation in the terminase M56 (glycine439 to arginine), the primase M70 (cysteine890 to tyrosine) and the alkaline nuclease M98 (proline324 to serine) [17]. These non-synonymous mutations were introduced individually and in combination into the MCMV K181 (Perth) variant virus using bacterial artificial chromosome (BAC) RecE/T homologous recombination, and showed that M56 and M98 individually did not contribute to the *ts* phenotype, but the M70 mutation alone and in combination with M56 and/or M98 rendered the virus *ts*, unable to replicate in mice and highly defective in DNA synthesis [18]. Experiments suggested that the M70 mutation produced a defective rather than an unstable protein, as replication was attenuated in Raw 264.7 macrophages at 37 °C, possibly reducing the amount of functional protein under different environmental conditions [19]. M70 is homologous to HCMV UL70 and a multiple alignment of herpesvirus UL70 primase homologues showed that the cysteine mutated in *tsm5* is conserved in all cytomegaloviruses, MCMV (K181, Smith), HCMV (human herpes virus 5, strain Merlin), monkey cytomegaloviruses (chimpanzee, rhesus), but not other herpesviruses [herpes simplex virus (HSV-1), human herpes viruses 3, 4, 6, 7 and 8 (HHV-3, HHV-4, HHV-6, HHV-7, HHV-8)] [18, 20]. It is located in close proximity (15 amino acids upstream) to the zinc finger motif and could, therefore, interfere with its function. Mutation of either one of the three cysteines and one histidine of the zinc finger rendered HCMV non-viable; thus, the zinc finger is essential for virus replication [21]. This suggested that the cysteine to tyrosine mutation may be an ideal candidate for attenuation of HCMV to produce a live attenuated vaccine. Unfortunately, this region of the primase appears to be particularly important for its function, as the tyrosine mutation had a marked effect on the protein structure and was, thus, very unstable and readily reverted to methionine or serine. A metagenomic analysis showed that these reversions produced a protein with a structure similar to that of the wt and the resultant viruses had a wt phenotype [19].

Sequencing of a number of immune evasion genes showed that no mutations were present in ORFs m04, m06, M33, M37, m38.5, m144, m152 or m157, although mutations were found in M27 (alanine568 to serine) and M36 exon1 (valine54 to isoleucine). When these mutations were introduced into K181 Perth virus together, the resultant virus was *ts* in mouse embryo fibroblast (MEF) cells and replication was drastically reduced in macrophages at 37 °C [22]. Thus, at least three of the polymorphic genes played a role in the attenuation of *tsm5*.

Interaction between mutations and their wt counterparts appears to maintain mutant populations, as in no virus stock produced over 18 years has wt virus emerged. Interestingly, several of the identified mutations in MCMV *tsm5* have homologues in HCMV (UL25, UL27, UL34, UL36, UL47, UL53, UL56, UL70, UL98). Thus, production of a mixture of similarly defined mutants of HCMV in which attenuating mutations in one or more mutants is counteracted by the wt nucleotide in other mutants could produce a stable live attenuated vaccine with similar properties to *tsm5* and the VZV vaccine virus.

### Preliminary studies changing codon preference

A number of studies have examined another approach to virus attenuation, namely introduction of less frequently used synonymous codons. All amino acids except methionine and tryptophan can be encoded by more than one synonymous codon. Codon manipulation has been used to enhance protein expression by changing codons to those most frequently used by the host [23]. With viruses, the codons used most frequently are generally those favoured by the host: termed ‘synonymous codon bias’. This was originally examined with poliovirus, where replacement of natural codons with synonymous non-preferred (minor) codons resulted in a significant reduction in virus yield and virus plaque sizes [24], with a consequent 100-fold reduction in virulence [25]. The high number of altered codons reduces the likelihood of reversion. This approach has been applied to a number of RNA viruses, but as far as I am aware not to a DNA virus [26]. Our working hypothesis was that replacement of preferred codons with those used less frequently by the mouse host would lower replicative fitness due to the paucity of tRNAs reducing the rate of translation (at the level of polypeptide synthesis) of the M70 primase, potentially disrupting viral DNA synthesis in infected cells.

Several strategies were examined for changing the codon sequence of the MCMV M70 ORF to those used less frequently by the mouse [19]. Initially, all codons that were not already used least frequently were changed to the least frequently used mouse codons. This resulted in an M70 ORF with a G+C content of 43 mol% compared to the K181 (Perth) ORF of 64 mol%, 403 of the 964 codons (42%) were changed. Whilst transfection of the modified BAC was successful in that fluorescence was detected for more than 12 days post-transfection, no viable virus could be isolated. To maintain the high G+C content evident in the MCMV genome, an alternative strategy was employed in which nine amino acids that have more than two codons were changed to the codon used sub-optimally by the mouse, but with the highest G+C content. This produced an M70 ORF with a G+C content of 66 mol% and a CpG of 379 compared to a CpG content of 381 for the K181 (Perth) ORF; 155 codons (16%) were changed. Transfection produced virus whose replication was similar to wt virus at 37 and 40 °C. Passage of the modified virus 10 times in tissue culture revealed that none of the altered codons had mutated. Interestingly, if the codon manipulation was performed for the tyrosine mutant version, no virus could be recovered but the revertant virus had the wt phenotype, suggesting that the 155 codon mutant was restricted.

## Conclusions

Work with MCMV has suggested two possible new approaches to a HCMV vaccine. Firstly, like the VZV vaccine, a mixture of mutants containing individual mutations in genes involved in DNA replication and immunoevasion could be used. Secondly, the use of altered codon preference in the carboxy terminus of the M70 (UL70) primase gene could be employed to produce a stable attenuated mutant. It would be interesting to examine these potential approaches with HCMV.
